# Albuminuria and markers for cardiovascular risk in 12-year-olds from the general Dutch population: a cross-sectional study

**DOI:** 10.1007/s00431-023-05152-4

**Published:** 2023-08-22

**Authors:** Valentina Gracchi, Sophie M. van den Belt, Eva Corpeleijn, Hiddo J. L. Heerspink, Henkjan J. Verkade

**Affiliations:** 1 Department of Pediatrics, Beatrix Children’s Hospital, University Medical Center Groningen, University of Groningen, P.O. Box 30.001 - CA13, 9700RB Groningen, The Netherlands; 2 Department of Clinical Pharmacy and Pharmacology, University Medical Center Groningen, University of Groningen, P.O. Box 30.001, 9700RB Groningen, The Netherlands; 3 Department of Epidemiology, University Medical Center Groningen, University of Groningen, P.O. Box 30.001, 9700RB Groningen, The Netherlands

**Keywords:** Albuminuria, Albumin-creatinine ratio, Children, Blood pressure, Cardiovascular risk, Epidemiology

## Abstract

In adults, albuminuria represents a risk factor for cardiovascular disease and is associated with hypertension and obesity. Pediatric data from the general population are inconsistent and largely based on randomly collected urine. A possible association between antenatal programming and albuminuria at school age has still to be investigated. The purpose of this study is to assess albuminuria in first morning void urine samples in a population-based pediatric cohort and to investigate cross-sectionally the association with factors related to cardiovascular risk. Moreover, we investigate the possible association of antenatal factors with albuminuria. A first morning void urine sample was collected in the population-based GECKO (Groningen Expert Center for Kids with Obesity) Drenthe cohort at the age of 12 years. We investigated cross-sectionally associations between albuminuria and body mass index (BMI), waist circumference (WC), blood pressure (BP) and antenatal factors. The prevalence of *U*_ACR_ (urinary albumin-creatinine ratio) ≥ 3 mg/mmol was 3.3% (95%CI 2.3–4.2). In a multivariate linear regression model, *U*_AC_ was negatively associated with z-BMI (*β*-0.08, *p* = 0.013) and positively with z-systolic BP (*β* 0.09, *p* = 0.006), model significance *p* = 0.002. *U*_ACR_ was negatively associated with z-BMI (*β* − 0.13, *p* < 0.001) and positively with z-diastolic BP (*β* 0.09, *p* = 0.003), model significance *p* = 0.001. Albuminuria was not significantly associated with antenatal factors such as gestational age and standardized birth weight.

*Conclusions*: Albuminuria in first morning void urine in 12-year-olds has a lower prevalence than previously reported by randomly collected samples. A negative association between albuminuria and BMI is confirmed. A positive association with blood pressure, but no association with antenatal factors was found.

**Table:** 

**What is known:** • While, in adults,* albuminuria is a recognized risk factor for cardiovascular disease and is associated with hypertension and obesity, pediatric data are inconsistent and largely based on randomly collected urine.* • *A possible association between antenatal programming and albuminuria at school age has still to be investigated.*	
**What is new:** • *In this population study on first morning void urine samples from 12-year-olds of the general population, albuminuria is negatively associated with body mass index, and positively associated with blood pressure, while there is no association with antenatal factors.* • *The prevalence of albuminuria at 12 years is lower than previously reported in studies based on randomly collected urine samples, probably due to elimination of orthostatic proteinuria.*

**Supplementary Information:**

The online version contains supplementary material available at 10.1007/s00431-023-05152-4.

## Introduction

Increased albuminuria is a well-known risk factor for the development of renal and cardiovascular disease in the adult population [1–7]. In the past decades, there has also been increasing evidence that albuminuria is associated with hypertension, obesity, and metabolic syndrome in adults [8–13].

In children, longitudinal data are lacking, and even the question whether albuminuria in children from the general population associates with the same markers for cardiovascular risk as in adults is still unanswered. In fact, the few epidemiological studies have been conducted on randomly collected urine, which can lead to an overestimation of albuminuria because of the high prevalence of orthostatic proteinuria in children [14–18]. In addition, data regarding the relationship between albuminuria and obesity in children are inconsistent [14, 16, 17, 19–21].

Little is also known about predisposing factors for albuminuria in children from the general population. Because many chronic diseases find their origin in the antenatal period, it has been hypothesized that also albuminuria could be determined by antenatal programming. Nevertheless, no association was found between antenatal or perinatal factors and albuminuria in toddlers, possibly because the children were still too young to express a renal phenotype of antenatal programming [22, 23].

The goal of this study was, therefore, to assess albuminuria in first morning void urine samples in a population-based cohort of 12-year-old children and to investigate cross-sectionally the association with factors related to cardiovascular risk, such as body mass index (BMI), waist circumference (WC), and blood pressure (BP). Moreover, we aimed to investigate the possible association of antenatal factors with albuminuria.

## Materials and methods

### Study population

This study was embedded in the Groningen Expert Center for Kids with Obesity (GECKO) Drenthe cohort, a population-based birth cohort with a focus on risk factors for childhood overweight. Detailed study design has been previously described and the cohort is registered at www.birthcohorts.net [24]. Briefly, all children born between April 2006 and April 2007 in the northern Dutch province of Drenthe could be included. Information on the pregnancy was obtained by questionnaires administered to the mothers in the third trimester of pregnancy. Information included maternal BMI before pregnancy, maternal smoking during pregnancy, and maternal education level. Data on delivery were recorded at birth, including gestational age, birth weight, placenta weight, maternal, and paternal age at delivery. Birth weight was standardized (std BW) for sex and gestational age using reference values from the 2019 updated Dutch Perinatal Registration (https://www.perined.nl) [25]. Anthropometric measurements were performed by specifically trained nurses of the municipal health services at the regular health control visits, taking place from birth up to the age of 10 years.

### Anthropometry and blood pressure at 10 years

At the 10-year control visit, height, weight, and waist circumference were measured. BMI (kg/m2) was calculated by dividing weight by height squared. BMI and WC were transformed into age- and sex-specific standardized z-scores, by using the Dutch Growth Analyser software (Growth Analyser, version 3.5, population data from 1997 as reference; Dutch Growth Research Foundation, Rotterdam, The Netherlands; available at https://www.growthanalyser.org) [26].

The 10-year health control visit was complemented by three consecutive measurements of arterial BP, performed by specifically trained pediatric nurses with an automated device (M3, Omron Healthcare Co, Japan) and appropriate cuff size. The BP was measured at the brachial artery, after 5 min of rest in sitting position. The mean of the three systolic and diastolic BP measurements was calculated and standardized for sex, age, and height using LMS (lambda-mu-sigma) box Cox transformations, using reference values from the 4th Report of the National High Blood Pressure Education Program (NHBPEP) [27].

### Urine collection and analysis

For the present study, we aimed to collect a first morning void urine sample from all children still actively participating in the GECKO Drenthe birth cohort at the age of 12 years. Between April 2018 and May 2019, parents received at home a kit with a plastic vial, an informed consent, and a collection form (for annotation of date and time of collection, as well as possible symptoms or previously diagnosed kidney diseases). Children were asked to collect a first morning void urine sample on a day that they had no symptoms (in particular, no fever, respiratory viral symptoms, or dysuria).

Directly upon urine delivery at the hospital, a urine dipstick was performed to rule out urinary tract infections and hematuria. Thereafter, urinary albumin concentration (*U*_AC_) was measured by immunoturbidimetric assay, with a lower limit of detection of 3.0 mg/L, and urinary creatinine concentration (*U*_CC_) by enzymatic assay, with a lower limit of detection of 0.1 mmol/L (both by Cobas® 8000 c502 analyzer, Roche Diagnostics, Germany). Urinary albumin-creatinine ratio (*U*_ACR_) was calculated by dividing *U*_AC_ by *U*_CC_ and expressed as mg of albumin per mmol of creatinine. Albuminuria was defined as *U*_AC_ > 20 mg/l and *U*_ACR_ ≥ 3 mg/mmol creatinine [28, 29].

The following exclusion criteria were applied: urine samples not collected as first morning void; urine samples collected more than 7 days before arrival at the hospital (because of uncertainty about stability of urine albumin at room temperature after a longer period of time); urine samples positive for hematuria on dipstick; children with suspected viral or urinary tract infections; incomplete informed consent form.

Written informed consent was obtained from both parents. Children who had already turned 12 at the time of urine collection also provided informed consent themselves, according to Dutch legislation. The study was approved by the Medical Ethics Committee of the University Medical Center Groningen, in accordance to the declaration of Helsinki of 1975, as revised in 1983.

### Statistical analysis

We reported characteristics of the study population as mean (standard deviation) or median (25th–75th percentile), as appropriate. The Mann–Whitney U test was used to test differences in *U*_AC_, *U*_ACR_, and *U*_CC_ between girls and boys. A possible relationship between *U*_AC_ or *U*_ACR_ and standardized z-scores for BMI, WC, BP, between *U*_AC_ or *U*_ACR_ and antenatal factors and between antenatal factors and standardized z-scores for BMI, WC, and BP was analyzed by univariate linear regression analysis. Candidate variables with a *p*-value < 0.10 in univariate analysis were selected for use in multivariate linear regression analysis with backward elimination. *U*_AC_ and *U*_ACR_ were log-transformed to account for their markedly right skewed distribution. A *p*-value < 0.05 was considered to be statistically significant. Data were analyzed using SPSS Statistics®, version 28.0 (IBM Corporation, Armonk, NY, USA).

## Results

### Population characteristics

**Fig. 1:**
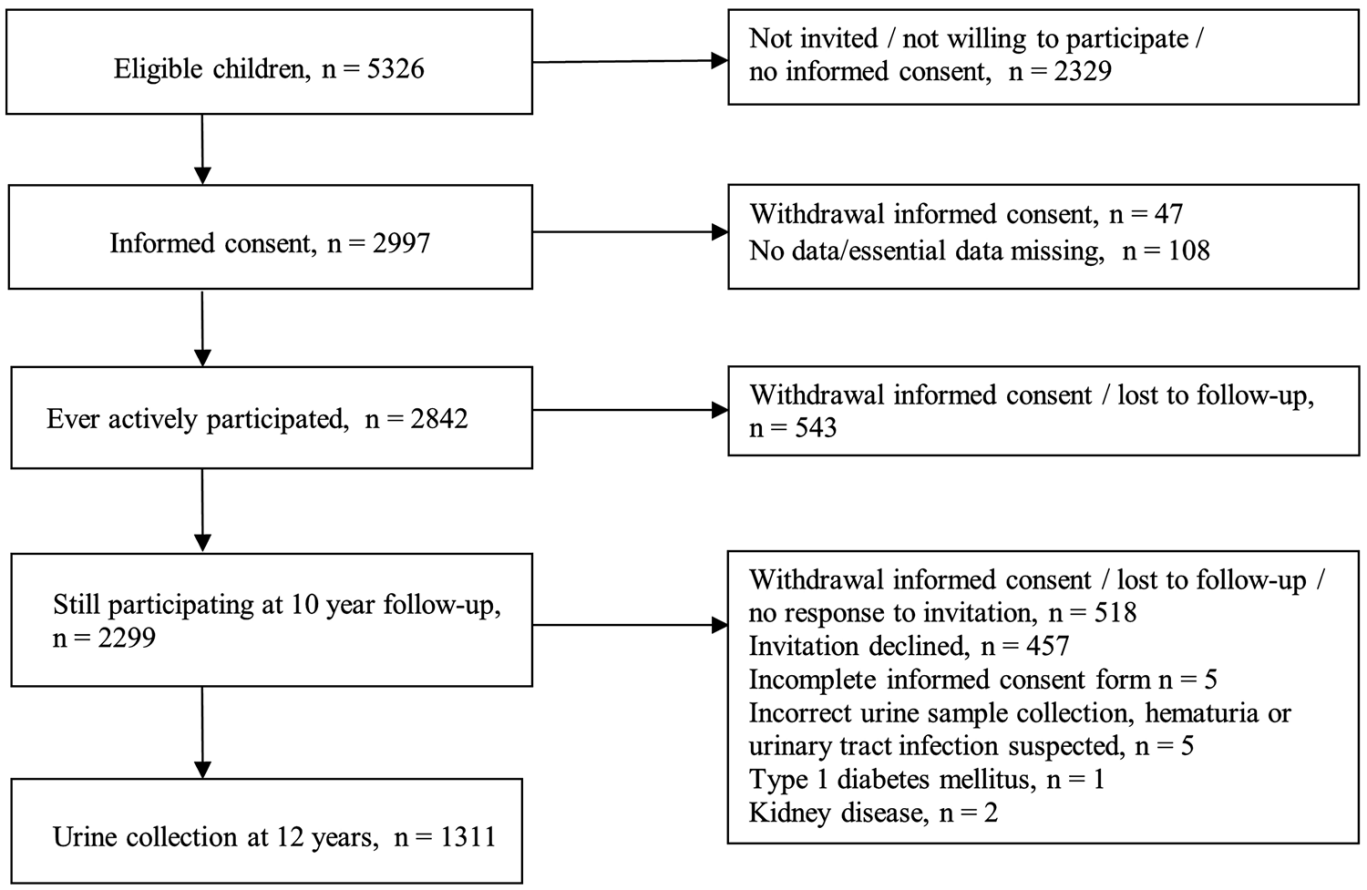
Flowchart of inclusion

Of the 2299 children still actively participating in the GECKO Drenthe cohort at the time of the 10-year follow-up, 1311 (57.0%) collected a first morning void urine sample at the age of 12 years (Fig. 1). The characteristics of the study population are shown in Table 1. Characteristics of the children who collected urine at 12 years as compared to the children who ever actively participated in the cohort but did not collect urine are shown in Supplementary Table S1. For the 1311 children who collected urine at 12 years of age, BMI was available for 1177 children, WC for 1026, and systolic and diastolic BP for 1110. As to the antenatal characteristics, information on gestational age at birth was present for 1295 children, birth weight for 1298, placenta weight for 870, maternal age at birth for 1309, paternal age at birth for 1254, maternal BMI before pregnancy for 1276, smoking during pregnancy for 1307, and data on maternal education for 1290. For all 1311 children with a urine collection, both *U*_AC_ and *U*_ACR_ were available for analyses.

**Table 1:** Characteristics of the study population

	**Children with** **12-year urine (*n* = 1311)**
Age at urine collection, years (25th–75th perc) Male sex, *n* (%) Height, cm (SD)	11.8 (11.6–12.1) 674 (51.4) 147.8 (6.8)
**Urinary measurements**	
* U*_AC_, mg/L (25th–75th perc)	4.5 (3.0^a^–8.7)
* U*_AC_ ≥ 20 mg/L, *n* (%)	103 (7.9)
* U*_CC_, mmol/L (25th–75th perc)	12.7 (9.5–16.2)
* U*_ACR_, mg/mmol (25th–75th perc)	0.4 (0.3–0.6)
* U*_ACR_ ≥ 3 mg/mmol, *n* (%)	43 (3.3)
**Cardiovascular risk factors**	
BMI, kg/m2 (25th–75th perc) z-BMI (SD)	17.1 (15.9–18.8) 0.18 (1.0)
WC, cm (25th–75th perc) z-WC (SD)	61.8 (58.7–66.5) 0.32 (1.0)
SBP, mmHg (SD) z-SBP (SD) DBP, mmHg (SD) z-DBP (SD)	107.7 (9.5) 0.28 (0.9) 63.4 (7.1) 0.11 (0.6)
**Antenatal characteristics**	
Gestational age, weeks (25th–75th perc)	40.0 (39.0–40.8)
Birth weight, g (SD) Std birth weight ^b^	3568 (559) 1.03 (0.1)
Placenta weight at birth, g (SD)	657 (152)
Maternal age at birth, years (SD)	31.9 (4.1)
Paternal age at birth, years (SD)	34.5 (4.8)
Maternal BMI before pregnancy, kg/m2 (25th–75th perc)	23.8 (21.5–26.6)
Maternal smoking during pregnancy, *n* (%)	134 (10.3)
Maternal education level: high, *n* (%) middle, *n* (%) low, *n* (%)	536 (41.5) 463 (35.9) 291 (22.6)

Values for continuous variables are reported as mean (standard deviation) or median (25th–75th percentiles), as appropriate; values for categorical variables as number (percentage). Reported age is age at time of urine collection. Anthropometric measurements have been performed at the 10-year follow-up visit

BMI (body mass index) available for 1177 children, WC (waist circumference) for 1026, SBP (systolic blood pressure), and DBP (diastolic blood pressure) for 1110. Information on gestational age at birth present for 1295 children, birth weight for 1298, placenta weight for 870, maternal age at birth for 1309, paternal age at birth for 1254, maternal BMI before pregnancy for 1276, smoking during pregnancy for 1307, and data on maternal education for 1290. All urinary variables available for 1311 children

*U*_AC_ urinary albumin concentration (^a^ 3 mg/L is the lower limit of detection), *U*_CC_ urinary creatinine concentration, *U*_ACR_ urinary albumin-to-creatinine ratio. Conversion factor for *U*_ACR_ mg/mmol: 0.113 mg/g

^b^ Std birth weight: birth weight standardized for sex and gestational age using reference values from the Dutch Perinatal Registration (https://www.perined.nl)

**Fig. 2:**
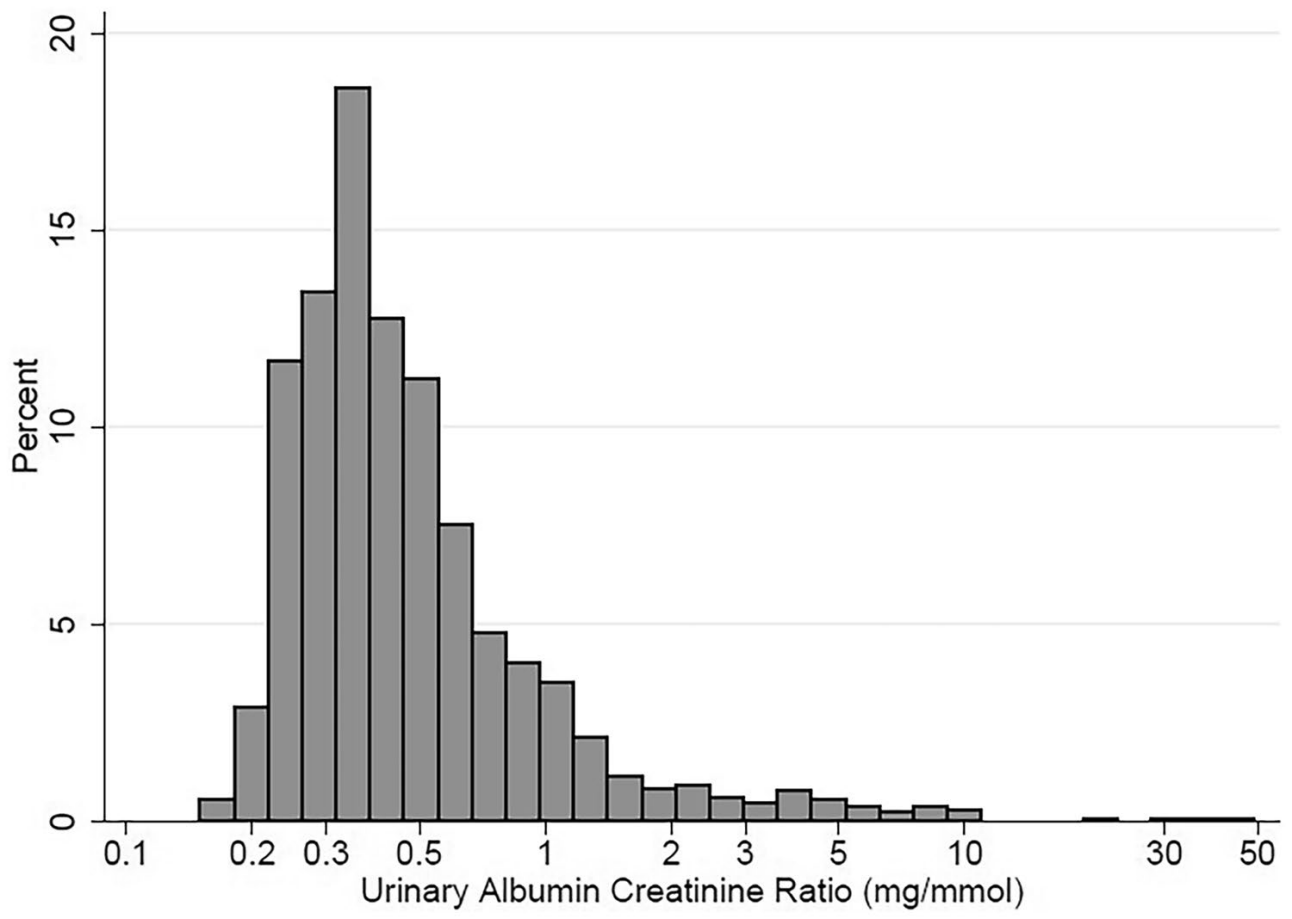
Distribution curve of *U*_ACR_ (urinary albumin-creatinine ratio) in the GECKO Drenthe cohort at 12 years

The prevalence of albuminuria based on *U*_AC_ ≥ 20 mg/L was 7.9% (95% CI 6.4–9.4). The prevalence of albuminuria based on *U*_ACR_ ≥ 3 mg/mmol creatinine was 3.3% (95% CI 2.3–4.2). Median *U*_AC_ and *U*_ACR_ were 4.5 mg/L (25th–75th percentile: 3.0–8.7 mg/L; 95th percentile: 29.3 mg/L) and 0.4 mg/mmol (25th–75th percentile: 0.3–0.6 mg/mmol; 95th percentile: 2.0 mg/mmol), respectively. Both *U*_AC_ and *U*_ACR_ were higher in girls than in boys (*p* = 0.02 for *U*_AC_; *p* < 0.001 for *U*_ACR_), while *U*_CC_ did not differ significantly between sexes (*p* = 0.10; Supplementary Table S2). The distribution of *U*_ACR_ is shown in Fig. 2.

### Association of urinary albumin with cardiovascular risk factors

We tested the hypothesis that urinary albumin at the age of 12 years is associated with factors related to increased cardiovascular risk (Table 2). In the multivariate linear regression model, *U*_AC_ was negatively associated with z-BMI (*β* − 0.08, *p* = 0.013) and positively with z-systolic BP (*β* 0.09, *p* = 0.006), with a significance of the model of *p* = 0.002. *U*_ACR_ was negatively associated with z-BMI (*β* − 0.13, *p* < 0.001) and positively with z-diastolic BP (*β* 0.09, *p* = 0.003), with a significance of the model of *p* = 0.001.

**Table 2:** Association of albuminuria with cardiovascular risk factors

	**Log U**_**AC**_			**Log U**_**ACR**_		
**Univariate linear regression model**						
	***R***^**2**^	**Std *β* (95% CI)**	***p*-value**	***R***^**2**^	**Std *β* (95% CI)**	***p*-value**
**z-BMI**	0.009	− 0.10 (− 0.19 to − 0.05)	< 0.001*	0.020	− 0.14 (− 0.28 to − 0.12)	< 0.001*
**z-WC**	0.005	− 0.07 (− 0.16 to − 0.01)	0.021*	0.011	− 0.10 (− 0.23 to − 0.06)	< 0.001*
**z-SBP**	0.006	0.08 (0.02–0.15)	0.008*	0.006	0.08 (0.02–0.17)	0.010*
**z-DBP**	0.003	0.05 (− 0.00 to 0.09)	0.069	0.020	− 0.14 (− 0.28 to − 0.12)	< 0.001*

**Multivariate linear regression with backward elimination**						
	**Sth *β* (95% CI)**		***p*-value**	**Sth *β* (95% CI)**		***p*-value**
**z-BMI**	− 0.08 (0.12 to − 0.01)		0.013*	− 0.13 (− 0.14 to − 0.05)		< 0.001*
**z-SBP**	0.09 (0.02–0.14)		0.006*			
**z-DBP**				0.09 (0.04–0.17)		0.003*
	*F*(2, 1003) = 6.51, adjusted *R*^2^ = 0.011		0.002*	*F*(2, 1003) = 12.4, adjusted *R*^2^ = 0.022		0.001*

Univariate and multivariate linear regression of log *U*_AC_ (the logarithm of urinary albumin concentration) and log *U*_ACR_ (the logarithm of urinary albumin-creatinine ratio) on *z*-scores of body mass index (BMI), waist circumference (WC), systolic blood pressure (SBP), diastolic blood pressure (DBP), *CI* confidence interval

* Statistically significant. Candidate variables with a *p*-value < 0.10 in univariate analysis were selected for use in the multivariate analysis

### Association of urinary albumin with antenatal characteristics

We investigated the possible association of antenatal characteristics with urinary albumin at the age of 12 years (Table 3). Male sex was negatively associated with both *U*_AC_ and *U*_ACR_ (*β* − 0.16, *p* < 0.001; *β* − 0.19, *p* < 0.001, respectively). All other investigated variables were not associated with *U*_AC_ nor *U*_ACR_.

**Table 3:** Association of antenatal factors with albuminuria

	**Log *U***_**AC**_			**Log *U***_**ACR**_		
**Univariate linear regression model**						
	***R***^**2**^	**Std *β* (95% CI)**	***p*-value**	***R***^**2**^	**Std *β* (95% CI)**	***p*-value**
Sex, male	0.010	− 0.16 (− 0.25 to − 0.07)	< 0.001*	0.019	− 0.19 (− 0.27 to 10.02)	< 0.001*
Gestational age, weeks	0.001	0.01 (− 0.01 to 0.04)	0.351	0.001	0.01 (− 0.01 to 0.04)	0.264
Std birth weight	0.000	0.01 (− 0.25 to 0.43)	0.598	0.001	0.03 (− 0.15 to 0.44)	0.342
Placenta weight at birth, g	0.000	0.00 (0.00–0.00)	0.675	0.000	0.00 (0.00–0.00)	0.691
Maternal BMI before pregnancy, kg/m2	0.001	0.00 (0.00–0.00)	0.322	0.000	0.01 (− 0.01 to 0.01)	0.709
Maternal age at birth, years	0.002	− 0.01 (− 0.02 to 0.00)	0.132	0.001	− 0.01 (− 0.02 to 0.00)	0.181
Paternal age at birth, years	0.001	0.01 (− 0.01 to 0.02)	0.188	0.000	0.00 (− 0.01 to 0.01)	0.607
Smoking during pregnancy (yes vs. no)	0.000	0.00 (− 0.14 to 0.15)	0.943	0.000	− 0.04 (− 0.17 to 0.09)	0.518
Maternal education level (low/middle vs. high)	0.001	0.028 (− 0.04 to 0.14)	0.309	0.002	0.04 (− 0.01 to 0.14)	0.105

Univariate linear regression of antenatal factors on log *U*_AC_ (the logarithm of urinary albumin concentration) and log *U*_ACR_ (the logarithm of urinary albumin-creatinine ratio), *Std birth weight* birth weight standardized for sex and gestational age using reference values from the 2019 updated Dutch Perinatal Registration (https://www.perined.nl). *BMI* body mass index, *CI* confidence interval.

* Statistically significant

We also analyzed the possible association of antenatal factors on parameters related to cardiovascular risk. A positive association was found between standardized birth weight, maternal BMI before pregnancy, and smoking during pregnancy on one side and both child’s z-BMI and z-WC on the other. A negative association was found between placenta weight and z-systolic BP and between male sex and z-diastolic BP (Supplementary Table S3).

## Discussion

In this population-based study, we investigated albuminuria in 12-year-olds from the general population and analyzed cross-sectionally if there were associations with cardiovascular risk factors and antenatal factors.

The novel contribution of this study is that albuminuria was determined in first morning void urine samples and not in randomly collected samples, in an age group known to have a high prevalence of orthostatic proteinuria.

Our data show that 12-year-old children have a wide range of albuminuria, as previously reported in toddlers from the same cohort and adults from the general adult population of the same geographical region [23]. General population cohorts with children in the same age range but from different geographical regions have previously also reported great variability in albuminuria but a higher prevalence of albuminuria, ranging between 8.9 and 15.1% [14–17]. This discrepancy can be at least partially explained by differences in protocols for urine collection, as the cited studies determined albuminuria in randomly collected urine and the authors recognized this as an important limitation for the interpretation of their results [14–17]. The higher reliability of first morning void urines for the estimation of daily albumin excretion has already been described [30]. Nevertheless, it is important to stress that its value might be even greater in children than in adults, as orthostatic proteinuria occurs in up to a fourth of children aged 11–18 years [18]. We are aware of only two studies on first morning void urines performed in children in the same age range as our study. Both studies have, in our opinion, important limitations such as an exclusion criterion of a positive dipstick test for proteinuria and the use of sole urinary dipsticks to define albuminuria [31].

Our study confirms a higher level of albuminuria in girls than in boys, in analogy to what was seen at the age of 2 years in the same cohort, and to other pediatric cohorts [14–17, 23, 32]. In the adult population, higher *U*_ACR_ levels in women than in men have been attributed to the lower muscle mass in women, leading to lower *U*_CC_ and therefore to higher *U*_ACR_. This seems not to apply to 12-year-old children because differences in *U*_ACR_ were driven by differences in *U*_AC_ and not in *U*_CC_, as shown here and by others [15]. Some authors have speculated that an explanation for this difference between sexes could be a higher prevalence of orthostatic proteinuria in girls than in boys [16]. This hypothesis is, however, not confirmed by our data. The reason for a difference between sexes has still to be elucidated but might reflect physiological differences at pre-pubertal age. The alternative hypothesis of albuminuria as a marker for higher cardiovascular and renal risk in females than in males is not supported by adult data, showing a higher risk for men than for women [33].

When analyzing the association between albuminuria and cardiovascular risk factors, we found that albuminuria was negatively associated with BMI. This finding is puzzling, when taking into account the positive association between albuminuria, obesity, and kidney disease reported in adults [5, 8, 9, 34]. Although pediatric studies show conflicting results, a negative relationship has already been reported in other general population cohorts [14, 16, 17, 19, 21, 32, 35]. Some authors have proposed orthostatic proteinuria as a possible confounder [14, 16]. Nevertheless, our data does not support this hypothesis. A possible explanation for the negative relationship between albuminuria and overweight could be a higher physical activity in children with a low BMI, as albuminuria can be caused by intense physical activity. Because we did not ask children to refrain from intense physical activity on the day prior to urine collection, our study cannot exclude this confounder.

The positive association between albuminuria and blood pressure in our study is in line with previously published data in adults from the general population and seems to confirm the role of albuminuria as a cardiovascular risk marker also in children [5–7, 13].

Antenatal factors were also investigated as possible risk factors for albuminuria. No association was found. Our findings confirm the previous reported lack of association between birth weight and *U*_ACR_ in Chinese school-aged children and add value to it because in our study children with proteinuria were not excluded [32]. However, it is important to mention that a negative association has been previously described for very low birth weight children [36]. Our interpretation is that such an association could be true for premature and low birth weight children solely, but not for the general population, at least at a medium-term follow-up.

The data from our cohort also allowed to explore antenatal factors that could be of influence on cardiovascular risk. This hypothesis was generated on the basis of the Barker’s hypothesis and the increasing recognition that the development of chronic diseases begins at a young age, probably even in the antenatal period [22]. Smoking during pregnancy was not associated with blood pressure, in analogy to the findings at the age of 5–6 years, and there was no association between maternal BMI before pregnancy or standardized birth weight and child’s blood pressure, in contrast with previous published data [37]. The association between higher birth weight and obesity has been described before [38]. In contrast to other studies, gestational age did not show a predictive value for blood pressure or obesity [39, 40]. Possibly this is due to a low number of small-for-gestational-age and premature children in our cohort.

The association of maternal BMI before pregnancy with higher BMI in children could be both interpreted as a constitutional or an environmental risk. Nevertheless, the positive association of smoking during pregnancy with child’s BMI suggests an environmental risk, with slimmer and non-smoking mothers having a healthier lifestyle that reflects on the offspring. Because environmental factors are modifiable, these data support the need to implement prevention policies, starting already from the antenatal period.

The most relevant strength of this study is the evaluation of albuminuria in first morning void urines. Other strengths of the study include a relatively large sample size, the exclusion of children with concomitant infections (a factor known to cause transient albuminuria), the measurement of urinary albumin in fresh (not frozen) urine samples by a single central laboratory, and the evaluation of albuminuria not only on the base of *U*_AC_ but also of *U*_ACR_ (as the latter corrects for urine dilution).

Our study has some methodological limitations. Due to the cross-sectional analysis of albuminuria and cardiovascular risk factors, it is not possible to establish causality or directionality of associations. Moreover, the lack of follow-up data does not allow to determine a possible impact of albuminuria at the age of 12 on cardiovascular risk in young adulthood. In addition, only half of the 12-year-olds in the cohort collected a first morning void urine. The well-known problem of sample attrition in cohort studies has possibly led to selection bias, as children who collected urine had a slightly lower WC during follow-up, older mothers with a higher education and less smoking in pregnancy.

There are also specific limitations regarding urine collection. The collection of a single urine sample can cause an overestimation of albuminuria, as a transient elevation of albumin excretion cannot be excluded. This can be due do different factors, including intense exercise and intercurrent infections. Although we attempted to limit this problem by instructing participants to collect urine early in the morning and not during infections, transient proteinuria could be an important confounder for our results.

In conclusion, albuminuria measured in a first morning void urine sample in 12-year-olds has a lower prevalence than previously reported in cohorts with randomly collected samples, probably due to elimination of orthostatic proteinuria. A negative association between albuminuria and BMI is confirmed. Moreover, a positive association with blood pressure but no association with antenatal factors was found. Further research is needed to assess the consequences of albuminuria at school age on cardiovascular risk factors in young adulthood.

### Supplementary Information

Below is the link to the electronic supplementary material.

Supplementary file1 (DOCX 41 KB)

## Data Availability

The datasets generated and analyzed during the current study are available from the corresponding author on reasonable request.

## Abbreviations

BMI: Body mass index
BP: Blood pressure
CI: Confidence interval
GECKO: Groningen Expert Center for Kids with Obesity
SD: Standard deviation
std BW: Standardized birth weight
*U*_AC_: Urinary albumin concentration
*U*_ACR_: Urinary albumin-creatinine ratio
*U*_CC_: Urinary creatinine concentration
WC: Waist circumference

## References

[1] Prognosis CKD, Matsushita K, van der Velde M, Astor BC, Woodward M, Levey AS, de Jong PE, Coresh J, Gansevoort RT (2010) Association of estimated glomerular filtration rate and albuminuria with all-cause and cardiovascular mortality in general population cohorts: a collaborative meta-analysis. Lancet 375:2073–2081 10.1016/S0140-6736(10)60674-5 PMC3993088 20483451

[2] Astor BC, Matsushita K, Gansevoort RT, van der Velde M, Woodward M, Levey AS, Jong PE (2011). Lower estimated glomerular filtration rate and higher albuminuria are associated with mortality and end-stage renal disease. A collaborative meta-analysis of kidney disease population cohorts. Kidney Int.

[3] Gansevoort RT, Matsushita K, van der Velde M, Astor BC, Woodward M, Levey AS, de Jong PE, Coresh J (2011). Lower estimated GFR and higher albuminuria are associated with adverse kidney outcomes. A collaborative meta-analysis of general and high-risk population cohorts. Kidney Int.

[4] van der Velde M, Matsushita K, Coresh J, Astor BC, Woodward M, Levey A, de Jong P (2011). Lower estimated glomerular filtration rate and higher albuminuria are associated with all-cause and cardiovascular mortality. A collaborative meta-analysis of high-risk population cohorts. Kidney Int.

[5] Atkins RC, Polkinghorne KR, Briganti EM, Shaw JE, Zimmet PZ, Chadban SJ (2004) Prevalence of albuminuria in Australia: the AusDiab Kidney Study. Kidney Int Suppl:S22–24 10.1111/j.1523-1755.2004.09206.x 15485411

[6] Jones CA, Francis ME, Eberhardt MS, Chavers B, Coresh J, Engelgau M, Kusek JW, Byrd-Holt D, Narayan KM, Herman WH, Jones CP, Salive M, Agodoa LY (2002). Microalbuminuria in the US population: third National Health and Nutrition Examination Survey. Am J Kidney Dis.

[7] Hillege HL, Janssen WM, Bak AA, Diercks GF, Grobbee DE, Crijns HJ, Van Gilst WH, De Zeeuw D, De Jong PE (2001). Microalbuminuria is common, also in a nondiabetic, nonhypertensive population, and an independent indicator of cardiovascular risk factors and cardiovascular morbidity. J Intern Med.

[8] Ferris M, Hogan SL, Chin H, Shoham DA, Gipson DS, Gibson K, Yilmaz S, Falk RJ, Jennette JC (2007). Obesity, albuminuria, and urinalysis findings in US young adults from the Add Health Wave III study. Clin J Am Soc Nephrol.

[9] Kim H, Kim HJ, Shin N, Han M, Park H, Kim M, Kwon H, Choi SY, Heo NJ (2014). Visceral obesity is associated with microalbuminuria in nondiabetic Asians. Hypertens Res.

[10] Ren M, Sun K, Li F, Qi YQ, Lin DZ, Li N, Li Y, Yan L (2016). Association between obesity measures and albuminuria: a population-based study. J Diabetes Complicat.

[11] Rashidbeygi E, Safabakhsh M, Delshad Aghdam S, Mohammed SH, Alizadeh S (2019). Metabolic syndrome and its components are related to a higher risk for albuminuria and proteinuria: evidence from a meta-analysis on 10,603,067 subjects from 57 studies. Diabetes Metab Syndr.

[12] Ritz E, Nowicki M, Fliser D, Hörner D, Klimm HP (1994). Proteinuria and hypertension. Kidney Int Suppl.

[13] Ren F, Li M, Xu H, Qin X, Teng Y (2021). Urine albumin-to-creatinine ratio within the normal range and risk of hypertension in the general population: a meta-analysis. J Clin Hypertens (Greenwich).

[14] Larkins N, Teixeira-Pinto A, Craig J (2017). The population-based prevalence of albuminuria in children. Pediatr Nephrol.

[15] Larkins NG, Kim S, Carlin JB, Grobler AC, Burgner DP, Lange K, Craig JC, Wake M (2019). Albuminuria: population epidemiology and concordance in Australian children aged 11–12 years and their parents. BMJ Open.

[16] Nguyen S, McCulloch C, Brakeman P, Portale A, Hsu CY (2008). Being overweight modifies the association between cardiovascular risk factors and microalbuminuria in adolescents. Pediatrics.

[17] Kim S, Macaskill P, Hodson EM, Daylight J, Williams R, Kearns R, Vukasin N, Lyle DM, Craig JC (2017). Beginning the trajectory to ESKD in adult life: albuminuria in Australian aboriginal children and adolescents. Pediatr Nephrol.

[18] Brandt JR, Jacobs A, Raissy HH, Kelly FM, Staples AO, Kaufman E, Wong CS (2010). Orthostatic proteinuria and the spectrum of diurnal variability of urinary protein excretion in healthy children. Pediatr Nephrol.

[19] Hirschler V, Molinari C, Maccallini G, Aranda C (2010). Is albuminuria associated with obesity in school children?. Pediatr Diabetes.

[20] Sanad M, Gharib A (2011). Evaluation of microalbuminuria in obese children and its relation to metabolic syndrome. Pediatr Nephrol.

[21] Sawamura LS, Souza GG, Santos J, Suano-Souza FI, Gessullo ADV, Sarni ROS (2019). Albuminuria and glomerular filtration rate in obese children and adolescents. J Bras Nefrol.

[22] Barker DJ (2007). The origins of the developmental origins theory. J Intern Med.

[23] Gracchi V, van den Belt SM, Kupers LK, Corpeleijn E, de Zeeuw D, Heerspink HJ (2016). Prevalence and distribution of (micro)albuminuria in toddlers. Nephrol Dial Transplant.

[24] L'Abée C, Sauer PJ, Damen M, Rake JP, Cats H, Stolk RP (2008). Cohort Profile: the GECKO Drenthe study, overweight programming during early childhood. Int J Epidemiol.

[25] Hoftiezer L, Hof MHP, Dijs-Elsinga J, Hogeveen M, Hukkelhoven C, van Lingen RA (2019). From population reference to national standard: new and improved birthweight charts. Am J Obstet Gynecol.

[26] Fredriks AM, van Buuren S, Burgmeijer RJ, Meulmeester JF, Beuker RJ, Brugman E, Roede MJ, Verloove-Vanhorick SP, Wit JM (2000). Continuing positive secular growth change in The Netherlands 1955–1997. Pediatr Res.

[27] National High Blood Pressure Education Program Working Group on High Blood Pressure in Children and Adolescents (2004). The fourth report on the diagnosis, evaluation, and treatment of high blood pressure in children and adolescents. Pediatrics.

[28] Levey AS, Eckardt KU, Dorman NM, Christiansen SL, Hoorn EJ, Ingelfinger JR, Inker LA (2020). Nomenclature for kidney function and disease: report of a Kidney Disease: Improving Global Outcomes (KDIGO) Consensus Conference. Kidney Int.

[29] Lambers Heerspink HJ, Brinkman JW, Bakker SJ, Gansevoort RT, de Zeeuw D (2006). Update on microalbuminuria as a biomarker in renal and cardiovascular disease. Curr Opin Nephrol Hypertens.

[30] Witte EC, Lambers Heerspink HJ, de Zeeuw D, Bakker SJ, de Jong PE, Gansevoort R (2009). First morning voids are more reliable than spot urine samples to assess microalbuminuria. J Am Soc Nephrol.

[31] Pugia MJ, Lott JA, Kajima J, Saambe T, Sasaki M, Kuromoto K, Nakamura R, Fusegawa H, Ohta Y (1999). Screening school children for albuminuria, proteinuria and occult blood with dipsticks. Clin Chem Lab Med.

[32] Wu D, Yang H, Luo J, Zhang G, Li S, Wang M, Tang X, Wang Z, Xu Z, Li Q (2014). Age- and gender-specific reference values for urine albumin/creatinine ratio in children of southwest China. Clin Chim Acta.

[33] Nitsch D, Grams M, Sang Y, Black C, Cirillo M, Djurdjev O, Iseki K, Jassal SK, Kimm H, Kronenberg F, Oien CM, Levey AS, Levin A, Woodward M, Hemmelgarn BR (2013). Associations of estimated glomerular filtration rate and albuminuria with mortality and renal failure by sex: a meta-analysis. BMJ.

[34] Chang AR, Grams ME, Ballew SH, Bilo H, Correa A, Evans M, Gutierrez OM (2019). Adiposity and risk of decline in glomerular filtration rate: meta-analysis of individual participant data in a global consortium. BMJ.

[35] Burgert TS, Dziura J, Yeckel C, Taksali SE, Weiss R, Tamborlane W, Caprio S (2006). Microalbuminuria in pediatric obesity: prevalence and relation to other cardiovascular risk factors. Int J Obes (Lond).

[36] Keijzer-Veen MG, Schrevel M, Finken MJ, Dekker FW, Nauta J, Hille ET, Frolich M, van der Heijden BJ, Dutch P-CSG (2005). Microalbuminuria and lower glomerular filtration rate at young adult age in subjects born very premature and after intrauterine growth retardation. J Am Soc Nephrol.

[37] Xie T, Falahi F, Schmidt-Ott T, Vrijkotte TGM, Corpeleijn E, Snieder H (2020). Early determinants of childhood blood pressure at the age of 6 years: The GECKO Drenthe and ABCD Study Birth Cohorts. J Am Heart Assoc.

[38] Boney CM, Verma A, Tucker R, Vohr BR (2005). Metabolic syndrome in childhood: association with birth weight, maternal obesity, and gestational diabetes mellitus. Pediatrics.

[39] Simmons RA (2013). Preeclampsia and prematurity as precursors to adolescent obesity. J Pediatr.

[40] Nuyt AM, Alexander BT (2009). Developmental programming and hypertension. Curr Opin Nephrol Hypertens.

