# Supplementary material for: Albuminuria and markers for cardiovascular risk in 12-year-olds from the general Dutch population: a cross-sectional study
Source: Eur J Pediatr. 2023 Aug 22;182(11):4921–9. doi: 10.1007/s00431-023-05152-4 (PMC10640422; doi:10.1007/s00431-023-05152-4)
Supplement: Supplementary file 1 — Supplementary file1 (DOCX 41 KB) [file 431_2023_5152_MOESM1_ESM.docx]

**Supplementary Table S1** Characteristics of children with and without urine collection at 12 years

|  | **Children with**  **12-years urine collection**  **(n = 1311)** | **Children without**  **12-years urine collection**  **(n = 1531)** | **p-value** |
| --- | --- | --- | --- |
| Male sex, n (%)  Height, cm (SD) | 674 (51.4)  147.8 (6.8) | 744 (48.6)  148.1 (6.9) | 0.134  0.306 |
| **Cardiovascular risk factors** | | | |
| BMI, kg/m2 (25th-75th perc) | 17.1 (15.9 - 18.8) | 17.4 (16.0 – 19.6) | <0.001* |
| WC, cm (25th-75th perc) | 61.8 (58.7 - 66.5) | 62.5 (59.0 – 68.4) | 0.007* |
| SBP, mmHg (SD)  DBP, mmHg (SD) | 107.7 (9.5)  63.4 (7.1) | 108.5 (9.9)  63.9 (7.8) | 0.662  0.060 |
| **Antenatal characteristics** | | | |
| Gestational age, weeks (25th-75th perc) | 40.0 (39.0 - 40.8) | 40.0 (38.8 – 40.8) | 0.336 |
| Birth weight, g (SD)  Std Birth weight ^a^ | 3568 (559)  1.03 | 3515 (536)  1.01 | 0.014*  0.124 |
| Placenta weight at birth, g (SD) | 657 (152) | 653 (153) | 0.630 |
| Maternal age at birth, years (SD) | 31.9 (4.1) | 30.3 (4.6) | <0.001* |
| Paternal age at birth, years (SD) | 34.5 (4.8) | 33.3 (4.9) | < 0.001* |
| Maternal BMI before pregnancy, kg/m2 (25th-75th perc) | 23.8 (21.5 - 26.6) | 23.6 (21.4 – 27.0) | 0.743 |
| Maternal smoking during pregnancy, n (%) | 134 (10.3) | 304 (19.6) | < 0.001* |
| Maternal education level: high, n (%)  middle, n (%)  low, n (%) | 536 (41.5)  463 (35.9)  291 (22.6) | 421 (30.4)  329 (23.8)  635 (45.8) | < 0.001* |

Values for continuous variables are reported as mean (standard deviation) or median (25th–75th percentile), as appropriate; values for categorical variables as number (percentage). Differences between groups tested by independent t-test, Mann-Whitney U test or χ^2^ test, as appropriate. * Statistically significant. ^a^ Std Birth weight: birth weight standardized for sex and gestational age using reference values from the Dutch Perinatal Registration, (<https://www.perined.nl>).

**Supplementary Table S2** Distribution of U_AC_, U_CC_ and U_ACR_ in the GECKO Drenthe cohort at the age of 12 years

| **Percentiles** | **U_AC_ (mg/L)** | | | **U_CC_ (mmol/L)** | | | **U_ACR_ (mg/mmol)** | | |
| --- | --- | --- | --- | --- | --- | --- | --- | --- | --- |
|  | **overall** | **boys** | **girls** | **overall** | **boys** | **girls** | **overall** | **boys** | **girls** |
| **5th** | 3.0 ^a^ | 3.0 ^a^ | 3.0 ^a^ | 6.3 | 6.4 | 6.1 | 0.2 | 0.2 | 0.2 |
| **25th** | 3.0 ^a^ | 3.0 ^a^ | 3.0 ^a^ | 9.5 | 9.8 | 9.1 | 0.3 | 0.3 | 0.3 |
| **50th** | 4.5 | 4.1 | 5.0 | 12.7 | 12.9 | 12.5 | 0.4 | 0.4 | 0.4 |
| **75th** | 8.7 | 7.7 | 9.8 | 16.2 | 16.4 | 15.9 | 0.6 | 0.6 | 0.6 |
| **95th** | 29.3 | 23.0 | 35.4 | 22.5 | 22.8 | 22.2 | 2.0 | 1.5 | 2.7 |

U_AC_, Urinary Albumin Concentration (^a^3 mg/L is the lower limit of detection); U_CC_, Urinary Creatinine Concentration; U_ACR_, Urinary Albumin-Creatinine Ratio.

Conversion factor for U_ACR_, mg/mmol : 0.113 = mg/g.

**Supplementary Table S3** Association of antenatal factors with cardiovascular risk factors.

|  | **z-SBP** | | | **z-DBP** | | | **z-BMI** | | | **z-WC** | | | |  |
| --- | --- | --- | --- | --- | --- | --- | --- | --- | --- | --- | --- | --- | --- | --- |
| **Univariate linear regression model** | | | | | | | | | | | | | | |
|  | **R^2^** | **Std β (95% CI)** | **p-value** | **R^2^** | **Std β (95% CI)** | **p-value** | **R^2^** | **Std β (95% CI)** | **p-value** | **R^2^** | **Std β (95% CI)** | **p-value** |  |  |
| Sex, male | 0.002 | -0.05 (-0.20 – 0.01) | 0.820 | 0.029 | -0.17 (-0.29 – -0.14) | <0.001* | 0.003 | 0.05 (-0.01 – 0.22) | 0.069 | 0.001 | 1.44 (0.60 – 2.29) | 0.389 |  |  |
| Gestational age, weeks | 0.000 | 0.00 (-0.99 – 1.09) | 0.053 | 0.000 | -0.00 (-0.267 – 1.47) | 0.790 | 0.000 | -0.01 (-0.03 – 0.39) | 0.872 | 0.000 | 0.01 (-0.35 – 0.42) | 0.861 |  |  |
| Std Birth weight^b^ | 0.000 | -0.02 (-0.56 – 0.26) | 0.480 | 0.000 | -0.19 (-0.39 – 0.20) | 0.530 | 0.034 | 0.18 (0.96 – 1.82) | <0.001* | 0.042 | 0.20 (1.08 – 1.99) | <0.001* |  |  |
| Placenta weight at birth, kg | 0.006 | -0.07 (-0.00 – 0.00) | 0.040* | 0.000 | 0.003 (-0.01 – 0.02) | 0.928 | 0.009 | 0.09 (0.00 – 0.01) | 0.009* | 0.022 | 0.15 (0.00 – 0.01) | <0.001* |  |  |
| Maternal BMI before pregnancy, kg/m2 | 0.002 | 0.043 (-0.00 – 0.21) | 0.156 | 0.003 | 0.04 (-0.00 – 0.02) | 0.060 | 0.106 | 0.32 (0.06 – 0.08) | <0.001* | 0.104 | 0.32 (0.06 – 0.09) | <0.001* |  |  |
| Maternal age at birth,  years | 0.000 | 0.02 (-0.01 – 0.20) | 0.545 | 0.002 | -0.04 (-0.16 – 0.00) | 0.176 | 0.001 | 0.32 (-0.01 – 0.22) | 0.270 | 0.000 | 0.19 (-0.10 – 0.02) | 0.536 |  |  |
| Paternal age at birth,  years | 0.001 | 0.03 (-0.01 – 0.19) | 0.275 | 0.001 | -0.03 (-0.01 – 0.00) | 0.289 | 0.002 | 0.04 (-0.00 – 0.02) | 0.155 | 0.001 | 0.02 (-0.00 – 0.02) | 0.462 |  |  |
| Smoking during pregnancy  (yes vs no) | 0.000 | -0.01 (-0.22 – 0.15) | 0.720 | 0.000 | -0.01 (-0.16 – 0.10) | 0.651 | 0.004 | 0.06 (0.02 – 0.41) | 0.028* | 0.008 | 0.09 (0.10– 0.52) | 0.005* |  |  |
| Maternal education level (low/middle vs high) | 0.001 | -0.2 (-0.15 – 0.06) | 0.425 | 0.001 | -0.04 (-0.13 – 0.03) | 0.205 | 0.004 | -0.10 (-0.33 – -0.10 | <0.001* | 0.015 | -0.12 ( -0.37 – -0.12) | <0.001* |  |  |
| **Multivariate linear regression with backward elimination** | | | | | | | | | | | | | | |
|  | **Std β (95% CI)** | | **p-value** | **Std β (95% CI)** | | **p-value** | **Std β (95% CI)** | | **p-value** | **Std β (95% CI)** | | **p-value** |  |  |
| Sex, male |  | |  | -0.18 (-0.31 – -0.15) | | <0.001* |  | |  |  | |  |  |  |
| Std Birth weight^b^ |  | |  |  | |  | 0.13 (0.46 – 1.50) | | <0.001* | 0.14 (0.56 – 1.66) | | <0.001* |  |  |
| Placenta weight at birth, kg | -0.07 (-0.00 – 0.00) | | 0.040* |  | |  |  | |  |  | |  |  |  |
| Maternal BMI before pregnancy, kg/m^2^ |  | |  |  | |  | 0.31 (0.05 – 0.08) | | <0.001* | 0.31 (0.05 – 0.09) | | <0.001* |  |  |
| Maternal age at birth, years |  | |  |  | |  |  | |  |  | |  |  |  |
| Paternal age at birth, years |  | |  |  | |  |  | |  |  | |  |  |  |
| Smoking during pregnancy  (yes vs no) |  | |  |  | |  | 0.13 (0.19 – 0.65) | | <0.001* | 0.14 (0.24 – 0.75) | | <0.001* |  |  |
| Model | F(1, 745) = 4.3, adjusted R^2^ 0.04 | | 0.04* | F(2, 1047) = 19.2, adjusted R^2^ 0.03 | | <0.001* | F(4, 764) = 37.0 adjusted R^2^ 0.12 | | <0.001* | F(3, 671) = 35.3, adjusted R^2^ 0.13 | | <0.001* |  |  |

Univariate and multivariate linear regression of antenatal factors on z-scores of Systolic Blood Pressure (SBP), Diastolic Blood Pressure (DBP), Body Mass Index (BMI) and waist circumference (WC); ^b^ Std Birth weight: birth weight standardized for sex and gestational age using reference values from the 2019 updated Dutch Perinatal Registration, (<https://www.perined.nl>). CI, Confidence Interval. * Statistically significant. Candidate variables with a p-value < 0.10 in univariate analysis were selected for use in the multivariate analysis
